# REV-ERBα Agonist SR9009 Promotes a Negative Energy Balance in Goldfish

**DOI:** 10.3390/ijms23062921

**Published:** 2022-03-08

**Authors:** Nuria Saiz, Lisbeth Herrera-Castillo, Esther Isorna, María Jesús Delgado, Marta Conde-Sieira, José Luis Soengas, Nuria de Pedro

**Affiliations:** 1 Fish Neuroendocrinology Group, Department of Genetics, Physiology and Microbiology, Faculty of Biology, Complutense University of Madrid, 28040 Madrid, Spain; nursaiz@ucm.es (N.S.); lisbethh@ucm.es (L.H.-C.); eisornaa@bio.ucm.es (E.I.); mjdelgad@bio.ucm.es (M.J.D.); 2 Centro de Investigación Mariña, Laboratorio de Fisioloxía Animal, Departamento de Bioloxía Funcional e Ciencias da Saúde, Facultade de Bioloxía, Universidade de Vigo, 36310 Vigo, Spain; mconde@uvigo.es (M.C.-S.); jsoengas@uvigo.es (J.L.S.)

**Keywords:** fish, *nr1d1*, feeding, body weight, growth, metabolism, glucose, lipids

## Abstract

REV-ERBα (*nr1d1, nuclear receptor subfamily 1 group D member* 1) is a transcriptional repressor that in mammals regulates nutrient metabolism, and has effects on energy homeostasis, although its role in teleosts is poorly understood. To determine REV-ERBα’s involvement in fish energy balance and metabolism, we studied the effects of acute and 7-day administration of its agonist SR9009 on food intake, weight and length gain, locomotor activity, feeding regulators, plasma and hepatic metabolites, and liver enzymatic activity. SR9009 inhibited feeding, lowering body weight and length gain. In addition, the abundance of *ghrelin* mRNA decreased in the intestine, and abundance of *leptin-aI* mRNA increased in the liver. *Hypocretin*, *neuropeptide y* (*npy*), and *proopiomelanocortin* (*pomc*) mRNA abundance was not modified after acute or subchronic SR9009 administration, while hypothalamic cocaine- and amphetamine-regulated transcript (*cartpt-I*) was induced in the subchronic treatment, being a possible mediator of the anorectic effects. Moreover, SR9009 decreased plasma glucose, coinciding with increased glycolysis and a decreased gluconeogenesis in the liver. Decreased triglyceride levels and activity of lipogenic enzymes suggest a lipogenesis reduction by SR9009. Energy expenditure by locomotor activity was not significantly affected by SR9009. Overall, this study shows for the first time in fish the effects of REV-ERBα activation via SR9009, promoting a negative energy balance by reducing energetic inputs and regulating lipid and glucose metabolism.

## 1. Introduction

REV-ERBα is a nuclear receptor belonging to the REV-ERB family, which comprises REV-ERBα and REV-ERBβ [1]. REV-ERBα inhibits the expression of a plethora of genes, competing with activating ROR proteins by binding to promoters with RORE/RevRE sites [1,2]. Moreover, REV-ERBα can bind to two adjacent ROREs and recruit the corepressors NCOR1 (nuclear receptor co-repressor 1) and HDAC3 (histone deacetylase 3) to repress transcription [3,4,5]. Apart from this direct regulation, REV-ERBα also regulates indirectly gene transcription by interacting with other transcription factors [3]. REV-ERBα’s physiological ligand is the porphyrin heme, a metabolite involved in processes such as cellular respiration, redox balance, and the synthesis of several hormones [6,7,8,9].

REV-ERBα plays key roles in the regulation of several physiological functions, such as energy balance and circadian rhythms [2,7,8]. Accordingly, most of the genes regulated by REV-ERBα are related to circadian and metabolic functions [10,11]. In mammals, REV-ERBα influences carbohydrate metabolism [11,12,13,14], adipogenesis and thermoregulation [1,8,15,16], lipid metabolism [11,12,17,18], cholesterol and bile acid metabolism [19], myogenesis [20], oxidative functions [21], and behavior [22,23], among other processes [1]. This is probably because most REV-ERBα targets are involved in the metabolism of lipids and lipoproteins, highlighting the importance of this receptor in this metabolic function [11]. REV-ERBα is involved in both the lipolytic and lipogenic pathways of mammals, increasing the hepatic expression of carnitine palmitoyltransferase 1 (CPT-1) and of the fatty acid transport protein 1, thus suggesting an increase in fatty acid oxidation [24]. On the other hand, REV-ERBα reduces the gene expression of lipogenic enzymes, such as fatty acid synthase (FAS) and stearoyl-CoA desaturase 1 [18,25]. Carbohydrate metabolism in mammals is also regulated by this nuclear receptor, since a chronic treatment with the agonist SR9009 induces the expression of the glycolytic enzymes hexokinase (HK) and pyruvate kinase (PK) in muscle, together with a reduction in plasma glucose levels [24]. REV-ERBα also suppresses gluconeogenesis by repressing the expression of enzymes phosphoenolpyruvate carboxykinase (PECPK), glucose 6-phosphatase (G6Pase), and fructose 1,6-biphosphatase (FBPase) in the liver, reducing glucose production [7,26].

Studies in rodent models have demonstrated that REV-ERBα is involved in other components of energy homeostasis beyond metabolism. On the one hand, REV-ERBα agonists decrease feeding and body weight (bw) [13,24], although effects over feeding can depend on the background energetic status [13]. On the other hand, energy expenditure seems to be increased by REV-ERBα activation, since the agonist SR9009 causes a rise in oxygen consumption in mammals, which in some cases is concurrent with a higher locomotor activity, but not always [13,21,24]. 

In fish, REV-ERBα sequence and structure are quite similar to those of other vertebrates [27], although there is scarce evidence of REV-ERBα’s role in teleosts. Some studies have implicated it in the circadian system. Thus, daily oscillations of *nr1d1* expression have been described in zebrafish (*Danio rerio*) [28,29,30], Nile tilapia (*Oreochromis niloticus*) [31,32], Chinese perch (*Siniperca chuatsi*) [33], Atlantic cod (*Gadus morhua*) [34], and goldfish (*Carassius auratus*) [35]. Moreover, REV-ERBα inhibits *bmal* expression in zebrafish [36] and goldfish (own unpublished results), in agreement with the known role of REV-ERBα as a repressor of the molecular clock transcription network [10]. As for the possible involvement of REV-ERBα in fish metabolism, an adipogenesis role has been reported in zebrafish [28]. 

Considering the growing significance of REV-ERBα as a key regulator of energy metabolism in mammals, and the limited knowledge in fish, the purpose of this work was to investigate the role of REV-ERBα activation on metabolism and energy homeostasis using the teleost *Carassius auratus,* a model species often employed for food intake and metabolism studies in teleosts [37]. Hence, the effects of acute or subchronic administration of REV-ERB’s agonist SR9009 on food intake, body weight, length, and mRNA abundance of some well-known feeding regulators such as NPY, hypocretin (orexin), POMC, CART, ghrelin, and leptin were assessed. Changes in locomotor activity after subchronic administration of SR9009 were also evaluated to investigate possible effects of REV-ERBα activation on energy expenditure. We also analyzed the effects of SR9009 treatment on metabolism by assessing metabolite levels in plasma and the liver, as well as activity of hepatic enzymes involved in glucose and lipid metabolism. 

## 2. Results

### 2.1. Effects of SR9009 on Food Intake, Growth, Feeding Regulators and Locomotor Activity

**Figure 1:**
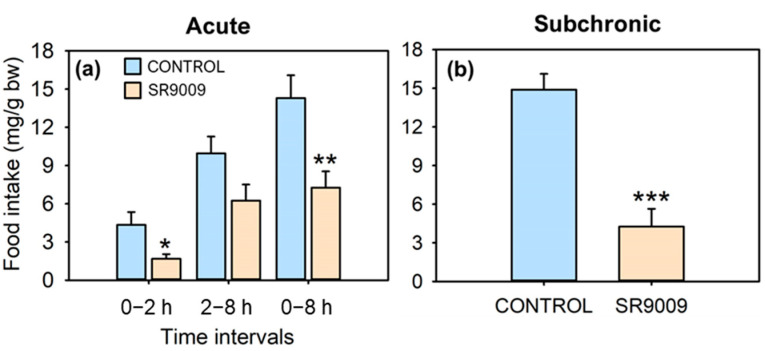
Effect of acute and subchronic SR9009 treatment on food intake in goldfish (*Carassius auratus*). (**a**) Food intake in the intervals of 0−2, 2–8, and 0–8 h after acute IP injection of vehicle alone (CONTROL) or containing SR9009 (100 µg/g bw, *n* = 9 fish/group). (**b**) Average daily food intake in the lapse of 2 h during subchronic (7 days) vehicle or SR9009 (100 µg/g bw) IP administration (*n* = 12 fish/group). Data are expressed as mean + S.E.M. * *p* < 0.05, ** *p* < 0.01, *** *p* < 0.001 SR9009 compared to control group (Student’s *t*-test).

**Figure 2:**
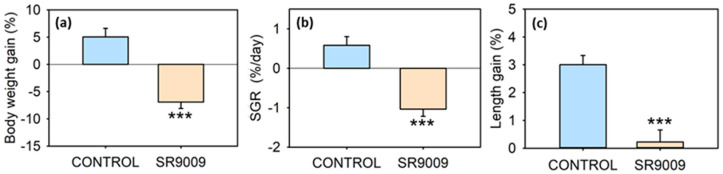
Effect of subchronic SR9009 treatment on growth of goldfish (*Carassius auratus*). Relative weight gain (**a**), specific growth rate (**b**), and relative standard length gain (**c**), over the 7 days of the subchronic IP administration of vehicle alone (CONTROL) or containing SR9009 (100 µg/g bw) (*n* = 12 fish/group). Data are expressed as mean + S.E.M. *** *p* < 0.001 SR9009 compared to control group (Student’s *t*-test).

Acute intraperitoneal (IP) administration of REV-ERBα’s agonist SR9009 caused a near-two-fold decrease in food intake in the lapses of 2 and 8 h post-injection (Figure 1a). In the subchronic treatment (7 days), average daily intake for 7 days also fell to less than a third compared with control fish (Figure 1b). The subchronic treatment with SR9009 strongly decreased body weight gain (Figure 2a) and specific growth rate (SGR, Figure 2b) to a point that they became negative (i.e., treated fish lost weight). Likewise, there were significant differences in fish length gain (Figure 2c), since control animals gained 3% in length while it remained almost unchanged in the SR9009 group. 

**Figure 3:**
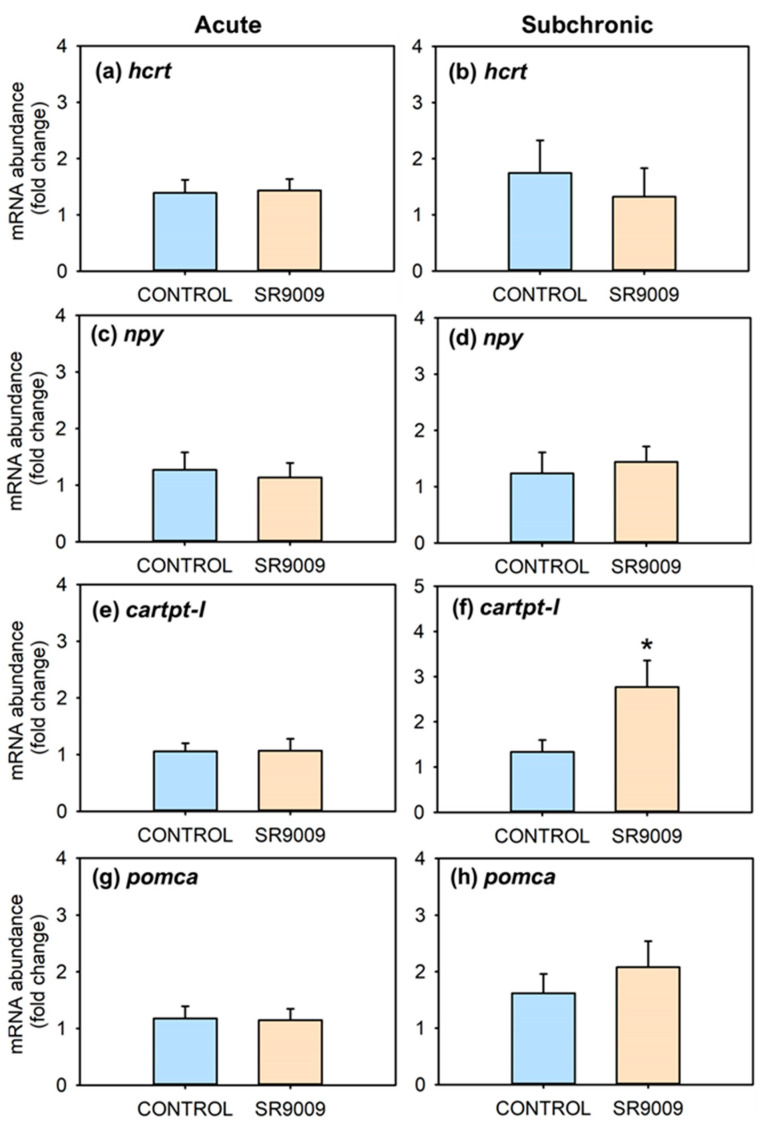
Effect of acute and subchronic SR9009 treatment on hypothalamic feeding regulators. Relative mRNA abundance of *hcrt* (**a**,**b**), *npy* (**c**,**d**), *cartpt-I* (**e**,**f**), and *pomca* (**g**,**h**) in the hypothalamus of goldfish (*Carassius auratus*) treated with vehicle alone (CONTROL) or containing SR9009 (100 µg/g bw). The left column shows results of the acute treatment (*n* = 9 fish/group), and the right column of the 7-day subchronic treatment (*n* = 12 fish/group). Data are expressed as mean + S.E.M. in relative units (2^−ΔΔCt^). * *p* < 0.05 SR9009 compared to control group (Student’s *t*-test).

**Figure 4:**
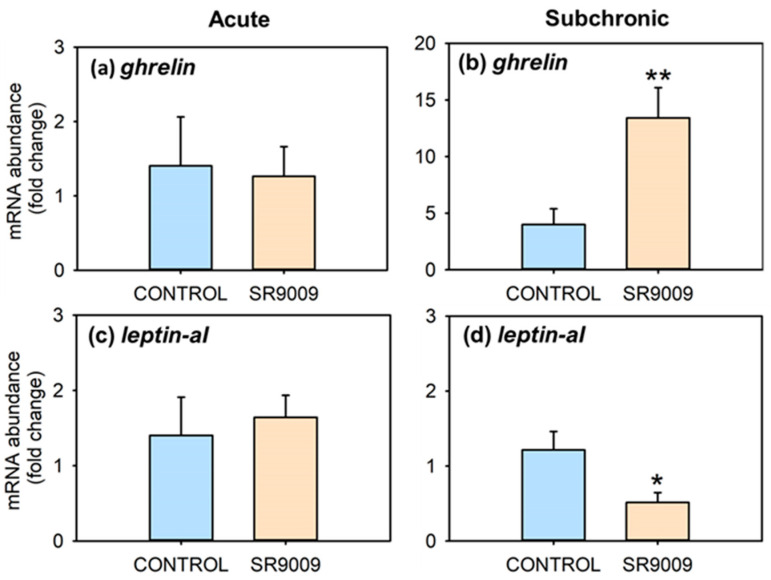
Effect of acute and subchronic SR9009 treatment on peripheral feeding regulators. Relative mRNA abundance of *ghrelin* (**a**,**b**) and *leptin-aI* (**c**,**d**) in the intestine and liver, respectively, of goldfish (*Carassius auratus*) treated with vehicle alone (CONTROL) or containing SR9009 (100 µg/g bw). The left column shows results of the acute treatment (*n* = 9 fish/group), and the right column of the 7-day subchronic treatment (*n* = 12 fish/group). Data are expressed as mean + S.E.M. in relative units (2^−ΔΔCt^). * *p* < 0.05, ** *p* < 0.01 SR9009 compared to control group (Student’s *t*-test).

Figure 3 summarizes the effects of acute or subchronic administration of SR9009 on mRNA abundance of hypothalamic feeding regulators. Fish treated acutely with this agonist showed no changes in the mRNA abundance of *hcrt*, *npy*, *cartpt-I*, and *pomc* (Figure 3a,c,e,g) at 3 h post-injection. The subchronic treatment with SR9009 increased hypothalamic mRNA abundance of *cartpt-I* (Figure 3f), without significant modifications in mRNA abundance of *hcrt*, *npy*, and *pomc* (Figure 3b,d,h). Regarding peripheral feeding regulators, intestinal mRNA abundance of *ghrelin* was distinctly increased, while *leptin-aI* mRNA values decreased in the liver in the SR9009 group after 7 days of treatment (Figure 4b,d), without statistically significant differences after acute injection of the REV-ERBα agonist (Figure 4a,c).

The locomotor activity was not significantly affected by the subchronic treatment with SR9009 (Figure S1), although there was a slight reduction in food-anticipatory activity (FAA) of treated fish. 

### 2.2. Effects of SR9009 on Metabolism

**Figure 5:**
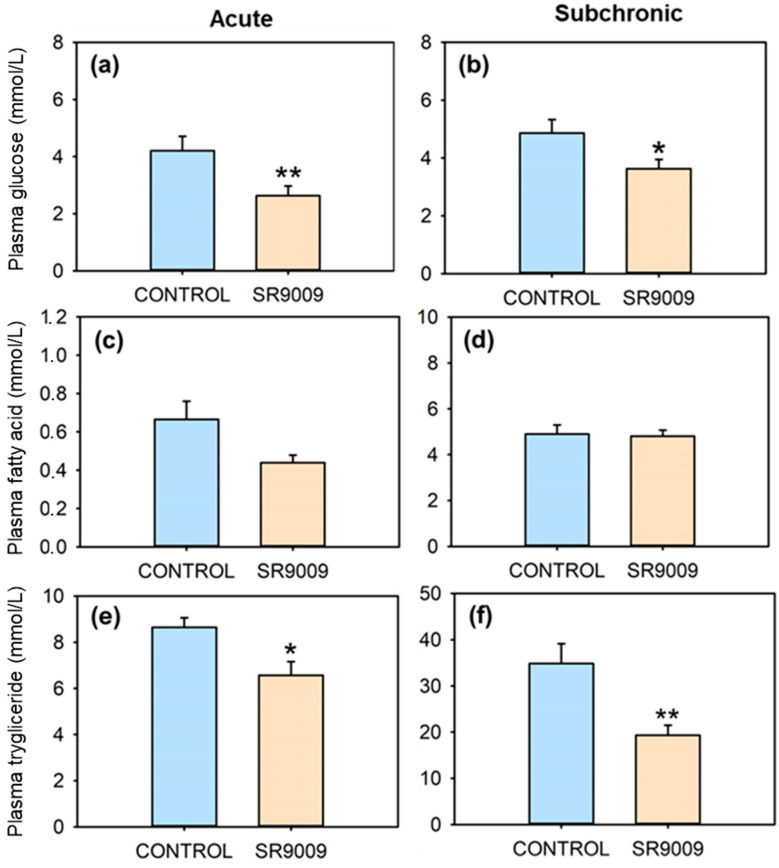
Effect of acute and subchronic SR9009 treatment on plasma levels of glucose (**a**,**b**), fatty acid (**c**,**d**), and triglyceride (**e**,**f**) in goldfish (*Carassius auratus*) treated with vehicle alone (CONTROL) or containing SR9009 (100 µg/g bw). Data are expressed as mean + S.E.M. (acute, *n* = 9/group; subchronic, *n* = 12/group). * *p* < 0.05, ** *p* < 0.01 SR9009 compared to control group (Student’s *t*-test).

**Figure 6:**
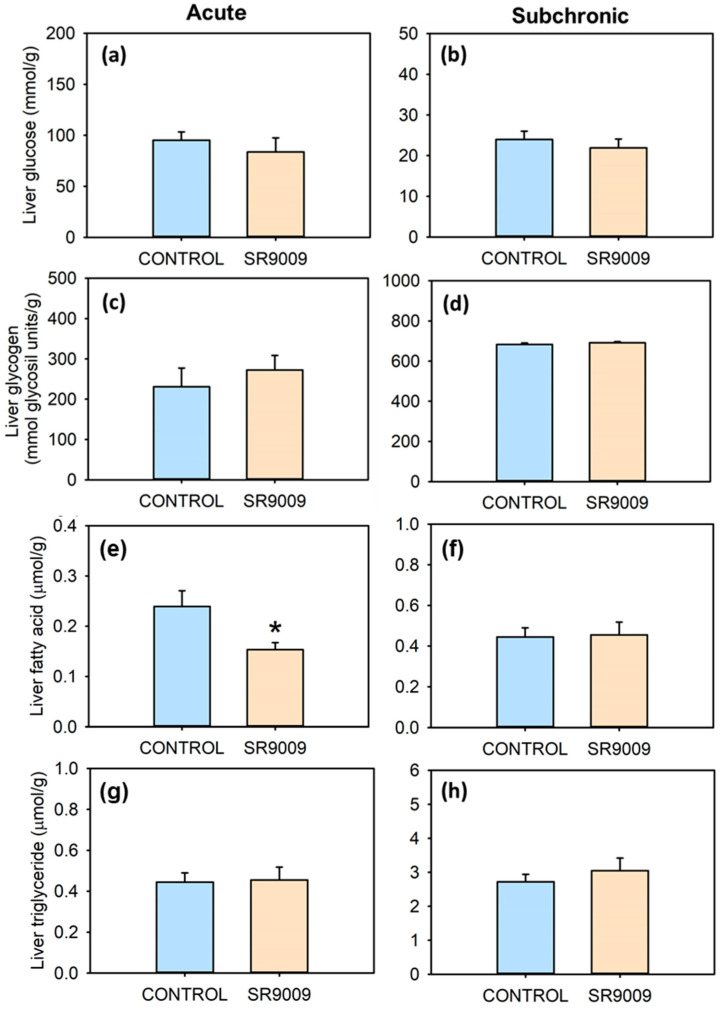
Effect of acute and subchronic SR9009 treatment on liver metabolites. Hepatic glucose (**a**,**b**), glycogen (**c**,**d**), fatty acid (**e**,**f**), and triglyceride (**g**,**h**) in goldfish (*Carassius auratus*) treated with vehicle alone (CONTROL) or containing SR9009 (100 µg/g bw). Data are expressed as mean + S.E.M. (acute, *n* = 9/group; subchronic, *n* = 12/group). * *p* < 0.05 SR9009 compared to control group (Student’s *t*-test).

SR9009 caused a significant reduction in plasma glucose (Figure 5a,b) and triglyceride levels (Figure 5e,f), after both acute and subchronic treatments. Plasma fatty acid levels remain unchanged (Figure 5c,d). No significant differences occurred in hepatic levels of triglyceride, glucose, or glycogen when comparing control and SR9009 groups (Figure 6). On the other hand, liver fatty acid concentration decreased in fish acutely treated with the REV-ERBα agonist compared with controls (Figure 6e). 

**Figure 7:**
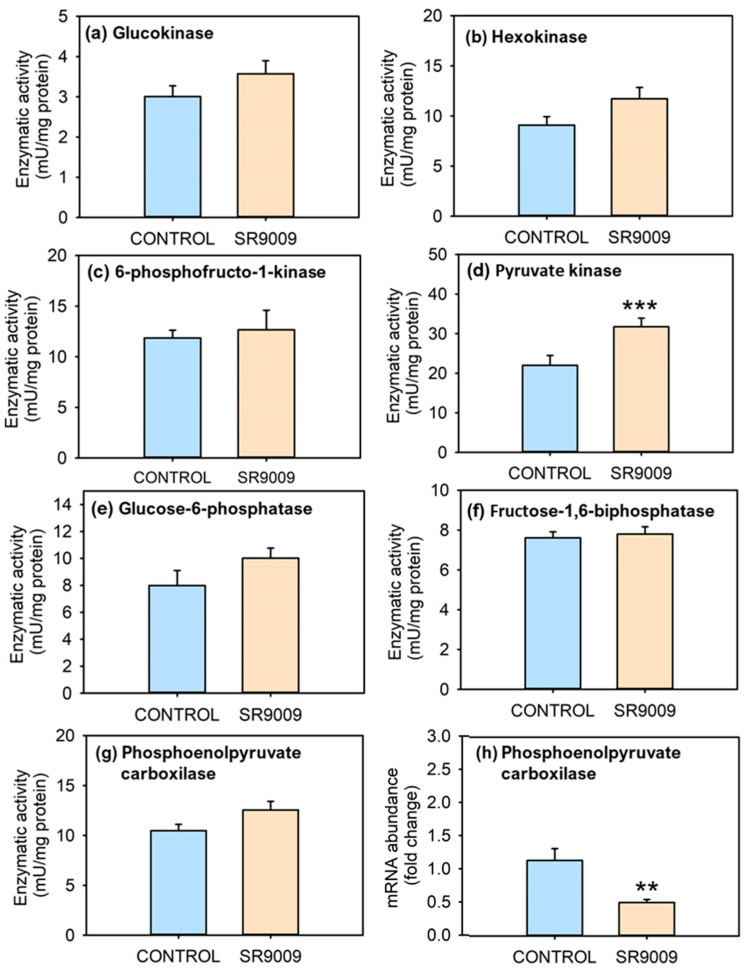
Effect of subchronic SR9009 treatment on enzymes related to glucose metabolism. Activity of enzymes related to glycolysis (**a**–**d**), glucose anabolism (**e**–**g**), and *pepck* mRNA abundance (**h**) in the liver of goldfish (*Carassius auratus*) treated with vehicle alone (CONTROL) or containing SR9009 (100 µg/g bw). Data are expressed as mean + S.E.M. (*n* = 12/group). ** *p* < 0.01, *** *p* < 0.001 SR9009 compared to control group (Student’s *t*-test).

Figure 7 outlines the effects of subchronic treatment with SR9009 over activities of hepatic enzymes related to glucose metabolism. In relation to enzymes involved in glycolysis, PK activity significantly increased in the liver of SR9009-treated fish (Figure 7d). The activity of remaining glycolytic enzymes did not show significant differences between control and SR9009-treated groups (Figure 7a–c); however, SR9009 tended to increase their activity, especially in HK and glucokinase (GCK). The subchronic IP treatment with SR9009 did not change enzymatic activity of: G6Pase, FBPase, and PEPCK (Figure 7e–g). However, *pepck* hepatic mRNA abundance was significantly reduced by SR9009 (Figure 7h). The activity of glucose-6-phosphate-dehydrogenase (G6PDH) was not modified by the subchronic treatment with SR9009 (Figure S2a). Similarly, glycogen synthase-GSase and phosphorylase-GPase were not significantly modified by the treatment with the REV-ERB agonist (Figure S2b,c).

**Figure 8:**
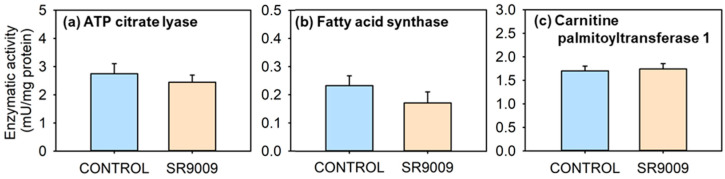
Effect of subchronic treatment SR9009 on the activity of enzymes related to lipid metabolism. Activity of ATP citrate lyase (**a**), fatty acid synthase (**b**), and carnitine palmitoyltransferase 1 (**c**) on the liver of goldfish (*Carassius auratus*) treated with vehicle alone (CONTROL) or containing SR9009 (100 µg/g bw). Data are expressed as mean + S.E.M. (*n* = 12/group).

The results obtained in parameters related to lipid metabolism are shown in Figure 8. Both lipogenic enzymes, especially ATP citrate lyase-ACLY (Figure 8a) and FAS (Figure 8b), showed slightly lower activities in fish treated subchronically with SR9009 than controls, particularly in FAS, whose reduction is close to the significance threshold. Lipolytic CPT-1 activity was not significantly modified in fish treated with SR9009 for 7 days (Figure 8c).

## 3. Discussion

The present results show that administration of REV-ERBα’s ligand SR9009 elicits strong effects over homeostatic regulation of food intake, body weight, and metabolism in goldfish, highlighting the role of this nuclear receptor as an important regulator of energy homeostasis in teleosts. 

Food intake is regulated by a central system, which integrates numerous neuroendocrine signals and works in combination with a peripheral system, mainly regulating satiety [38,39,40]. These feeding-regulating mechanisms are well conserved in fish and mammals. In the case of the nuclear receptor REV-ERBα, present results in goldfish point to a potent anorectic action of this nuclear receptor, since a drastic (up to a 75%) decrease in food intake occurred after acute and subchronic treatment with the agonist SR9009. The slight decrease in FAA (locomotor activity increase anticipating the predictable time of food arrival) observed after subchronic SR9009 suggests that this agonist could also act by decreasing initial phases of eating behavior, such as arousal and/or appetitive phases, as is also the case with other anorectic regulators in goldfish [41]. The fact that this anorectic effect was not mitigated along the subchronic treatment excludes possible tolerance phenomena in the experimental period (7 days), in agreement with low probability of developing short-term tolerance in response to the SR9009 regular administration observed in mice [42]. Studies in mammals vary from showing no changes in food intake after SR9009 chronic treatment in an obese mouse model [24], to showing that REV-ERBα agonist increases feeding in fasted rats and decreases it when they are free-fed [13]. In the present experiments, food intake was measured during the usual feeding time of the fish, with no fasting beyond maintenance conditions, and in the chronic treatment food was offered in excess amount, which could explain why present results resemble those seen in ad libitum-fed rats. 

The strong turn to a negative energy balance due to the anorectic effect of SR9009 is probably what caused the reduction in *leptin-aI* and increase in *ghrelin* mRNA abundance found in goldfish under the subchronic SR9009 treatment, aligning with decreases in *leptin-aI* and increases in *ghrelin* expression induced by 7-days’ fasting in fish [43]. These modifications in hepatic leptin and intestinal ghrelin would be a consequence of decreased food intake and weight in SR9009-treated fish, and do not seem to be directly involved in the anorectic action of this agonist. If this was the case, we would have expected an increase in leptin and a decrease in ghrelin, considering their respective anorexigenic and orexigenic role in vertebrates, including fish [38,39,40,44].

In mammals, hypothalamic neuropeptides are involved in the anorectic action of SR9009 through decreased brain mRNA abundance of the orexigenic peptide *hcrt* and its receptors after acute and chronic treatments [42]. In the present study, slight decrease in the hypothalamic mRNA abundance of *hcrt* in subchronic treated goldfish was observed, which alone could not justify the strong anorectic effect of SR9009. These different results in goldfish and mice may be due to species-specific differences and/or to differences in the experimental design. In the mammalian study, mice received two daily injections of the SR9009 agonist for 10 days, while a single daily injection for 7 days was performed in goldfish. Furthermore, in mice the effects occurred 6 h after acute or chronic injection of the agonist, while in fish they occurred 3 h post-injection in the acute experiment, and 24 h post-injection in the subchronic experiment. As for *npy* and *pomca* transcripts, no effects of SR9009 treatment occurred both in mammals and fish. The strong induction of the mRNA abundance of the anorectic neuropeptide *cartpt-I* suggests that the REV-ERB-induced reduction in food intake could be mediated by modifications in this hypothalamic neuropeptide. It is interesting to point out that leptin, which is the most-known inducer of CART [44,45,46], is underexpressed in fish of the SR9009 group, so other regulators such as insulin could be mediating the increase in CART [47]. Accordingly, some studies associate REV-ERBα activation with heightened insulin secretion and β-cell proliferation [14].

Conversely, the decreased feeding observed in the acute treatment was not associated with changes in the mRNA abundance of any of the food intake-regulating signals studied. These results allow us to suggest the involvement of changes in their receptors or in their plasmatic or cerebral levels. An alternative explanation for this anorectic effect might relate to the possible involvement of the hedonic system [22,48,49].

Fish weight decreased 7% and their length gain was near to null in fish treated with the agonist, while control animals gained weight and length, in agreement with reports in mice [24]. The food-intake reduction in goldfish injected with SR9009 does not seem to be the only cause of the decline in weight and growth observed in this experiment, since these reductions were more pronounced than what is usually seen in fasting goldfish [44]. Muscle mass was probably not the main reason for the body weight reduction, since SR9009 treatment overturns the loss of muscle mass in mice lacking REV-ERBα [50]. Instead, weight loss generated by SR9009 in mice is associated with a decrease in fat mass [24]. Another possibility is that a heightened energy expenditure (basal oxygen consumption and/or physical activity) might contribute to weight loss. In fact, SR9009 increased oxygen consumption in mice, although different locomotor responses were reported [21,24]. Depending on the energy status, opposite responses on locomotor activity have also been found after acute injection with a REV-ERBα agonist: a reduction in fasted rats versus an increase in fed rats [13]. Recordings show similar locomotor activities in both experimental groups, but a possible increase in oxygen consumption may be concurring in SR9009-treated goldfish. Additional studies are required to elucidate whether SR9009 also increases energy expenditure by this mechanism in fish, thus contributing to weight reduction.

Another important aspect related to energy expenditure pertains to changes in energy metabolism, especially in tissues such as the liver. Therefore, we assessed if SR9009 treatment affected the hepatic metabolism of lipids and carbohydrates in goldfish. Regarding carbohydrate metabolism, SR9009 lowered levels of plasma glucose of fish in both subchronic and acute treatments, a hypoglycemic effect similar to that found in mice [24,51]. This effect does not seem to be produced by reduced food intake in treated fish, since glucose levels also decreased after acute treatment, in which fish were not fed from the day before the injection onwards. The lower glycaemia was associated with a decreased hepatic mRNA abundance of the gluconeogenic enzyme *pepck*. PEPCK is considered one of the main rate-limiting enzymes in gluconeogenesis, eliciting robust effects over glucose levels [52]. In vitro and in vivo studies evidence that pharmacological activation of mouse REV-ERBα represses hepatic *pepck* to lower plasma glucose, probably due to REV-ERBα direct binding to a RevRE site [7,51]. However, we observed no evidence of decreased activity of this enzyme in the SR9009 group, which could suggest that the downregulation of enzymatic activity takes a longer time to be evident. To our knowledge, there are no previous studies in mammals regarding impact of SR9009 on enzymatic activity (available studies just described changes in mRNA abundance). Hepatic activity of other enzymes related to gluconeogenesis such as G6Pase and FBPase were unaffected in goldfish, in agreement with the absence of changes in the expression of both enzymes described in mouse hepatoma cells cultured with SR9009 [51]. However, a REV-ERBα repressor effect over G6Pase has been shown in liver cells treated with hemin [7]. Another possible cause related to lower glycaemia is the role of REV-ERBα in the endocrine pancreas, increasing insulin secretion [26]. The significant increase in the activity of PK, together with the slight raise in other glycolytic enzymes (GCK, HK, and 6-phosphofructo-1-kinase-PFK) in SR9009 group, suggests an enhancement of glycolytic potential, which could also contribute to the lower glycaemia, supporting also changes observed in *pepck* mRNA abundance, considering that both pathways are antagonistic. These results are also in agreement with studies in mice in which HK and PK expression levels in muscle are increased by treatment with the same agonist, increasing glucose oxidation [24].

A hypotriglyceridemic effect of REV-ERBα agonist has been reported in mammals [24]. Interestingly, in this work plasma triglyceride also fell in the acutely and subchronically SR9009-treated goldfish. This could relate to changes in the activity of enzymes involved in lipogenesis or lipolysis, as observed in mammals. Thus, *cpt-1* mRNA abundance in mouse muscle increased after REV-ERBα agonist treatment, suggesting an enhancement of fatty acid β-oxidation [24]. On the other hand, results in mammals point to a repression of several lipogenic genes in the liver by REV-ERBα [18,25]. In the present study, although no changes occurred in CPT-1 activity in the goldfish liver, the activities of the lipogenic enzymes FAS and ACLY were slightly reduced by SR9009 treatment. In mammals, this lower lipogenic potential induced by REV-ERBα appears to be related to daily rhythms of this nuclear receptor. Thus, in rodents, high levels of REV-ERBα occur during the inactive/fasting phase, reducing lipogenesis. Once REV-ERBα is depleted and the animal is active and feeding, lipid synthesis takes place again [18]. In agreement with this model, a daily rhythm in *nr1d1* mRNA abundance has been observed in the goldfish liver, with high levels also when inactive and fasting [35], which could lead to a lipogenesis reduction, as supported by REV-ERB-induced hypotriglyceridemia. In this direction, REV-ERBα is also known to repress the expression of apolipoprotein CIII, an important mediator of triglyceride metabolism whose loss has been associated with reduced levels of plasma triglyceride in mammals [17]. Thus, REV-ERBα activation seems to reduce circulating triglycerides in fish as in mammals, but the mechanisms are not yet well-defined in fish.

Although SR9009 has traditionally been used within in vivo studies as a target of REV-ERBα [21,50,51], there are a number of considerations to be taken into account. On the one hand, SR9009 is a dual REV-ERB agonist (REV-ERBα and REV-ERBβ) and we cannot rule out effects on REV-ERBβ receptor. Nevertheless, both nuclear receptors have overlapping functions, since both REV-ERBs appear to share many of the same target genes, and in addition REV-ERBα represents the dominant *rev-erb* paralog in the liver [2,4]. On the other hand, a recent study on cell proliferation suggested that SR9009 has REV-ERB-independent effects, although mechanisms involved remain to be determined [53]. Furthermore, food composition strongly affects levels of plasma metabolites and growth, so results might vary with a different diet [54]. Overall, this study assessed for the first time in fish the impact of a REV-ERBα agonist on several components of energy homeostasis. SR9009 decreases food intake, probably through the involvement of enhanced *cartpt-I* levels. Accordingly, it causes a strong reduction in body weight and length gain, probably provoking an increase in *ghrelin* and a decrease in *leptin-aI* mRNA abundance. As for intermediary metabolism, SR9009 inhibits gluconeogenic potential by repressing *pepck* and promotes glycolysis by an increased PK activity, resulting in low blood glucose. Reduction in plasma levels of triglyceride agrees with a reduction trend in the liver ACLY and FAS activities, suggesting a decrease in lipogenesis. As a whole, the present study displays a vision of the actions of REV-ERBα agonist SR9009 in fish energy homeostasis, promoting a negative balance by reducing food intake and modifying hepatic metabolism of lipids and carbohydrates. Further studies are necessary to fully characterize underlying mechanisms and assess the involvement of the circadian system in this regulation.

## 4. Materials and Methods 

### 4.1. Animals and Housing

Goldfish juveniles acquired from a local commercial supplier (ICA, Madrid, Spain) were kept in 60 L tanks with filtered and aerated freshwater (21 ± 1 °C, >90% O_2_ saturation). Water conditions were: nitrites 0.5 mg/L, pH 6–7, and marine salt (Neomarine, Brightwell Aquatics, AL, USA) 0.26 g/L. Standard maintenance conditions were at 12L:12D photoperiod (lights-on at 8 a.m.), and daily feeding at 10 a.m. with food pellets (1.5% bw; Sera Pond Biogranulat, Heinsberg, Germany) by automatic feeders. All fish used in the experiments described in the present study were considered healthy, with no observable lesions or obvious alterations in behavior. All proceedings complied with the Guidelines of the European Union Council (UE63/2010) and the Spanish Government (RD53/2013) for the use of animals in scientific proposals, and were approved by the Community of Madrid (PROEX 170.6/20).

### 4.2. SR9009 Administration

SR9009 (Sigma Chemical, Madrid, Spain) was dissolved in a vehicle containing 15% Kolliphor^®^ and 85% teleost saline solution (600 mg NaCl and 15.8 mg NaHCO_3_ in 100 mL distilled water). Fish were anesthetized in buffered tricaine methanesulfonate (MS222, 0.14 g/L, Sigma Chemical, Madrid, Spain) before being handled and injected to minimize stress. Intraperitoneal injections were performed using plastic 1 mL syringes and 0.3 mm needles, introduced near the ventral midline, immediately posterior to pelvic fins. Fish were injected with 10 µL/g bw of vehicle alone or containing SR9009 (100 µg/g bw). Injected fish were released back into the experimental tanks, where, in the absence of anesthetic, equilibrium and locomotor activity recovered within 1–2 min.

### 4.3. Experimental Designs 

#### 4.3.1. Acute Effect of SR9009 on Food Intake 

A first set of fish (13.7 ± 0.73 g bw) were IP injected (*n* = 9) at the scheduled feeding time (10 a.m., 24 h of fasting) with either vehicle alone or containing SR9009 (100 µg/g bw) as described above. After this, fish were individually placed in 5 L aquaria and food intake was quantified during the intervals of 0–2, 2–8, and 0–8 h post-injection, as described elsewhere [44]. In short, pre-weighed food was supplied in excess (3% bw, Sera Pond Biogranulat, Heisenberg, Germany), 10 min post-injection, and any remaining food was collected after 2 and 8 h and dried at 55 °C for 24 h. Food intake was calculated following the formula: FI=(Wi−Wf)×f, where Wi and Wf are the initial and remaining dry food weight, and f is the dilution factor [44].

Before this, the vehicle itself was tested in the same way against plain saline solution, and no significant effect was found on food intake (Figure S3).

#### 4.3.2. Acute Effect of SR9009 on Intake Regulators and Metabolism 

Goldfish (12.57 ± 0.72 g bw) were divided in two groups (*n* = 9/group) that were IP injected with vehicle or SR9009 (100 µg/g bw) at 10 a.m. (24 h of fasting). At 3 h post-injection, fish were anesthetized with MS-222 (0.14 g/L) and blood was sampled from the caudal vein and immediately centrifuged. After this, fish were sacrificed by anesthetic overdose (MS-222, 0.3 g/L) followed by spinal section. Then, the hypothalamus, liver, and intestine were sampled and kept at −80 °C, while plasma was stored at −20 °C until analysis. 

#### 4.3.3. Effect of Subchronic SR9009 Administration on Feeding, Growth, Locomotor Activity, and Metabolism

A total of 24 fish of 15.5 ± 0.53 g bw were divided into four tanks (6 fish/tank), two for each experimental group (control or SR9009, *n* = 12 fish/group). During the 7 experimental days, fish were fed at 10 a.m. at 3% bw, and remaining food was retrieved to quantify food intake at 2 h afterwards. At 1 p.m., fish were anesthetized, weighed, measured, and IP injected with vehicle or SR9009 (100 µg/g bw). Locomotor activity was also registered through the experimental period. On the eighth day (24 h post-injection and 27 h of fasting), fish were anesthetized to obtain blood from the caudal vein, and sacrificed by anesthetic overdose followed by spinal section. Blood was immediately centrifuged after extraction. The liver, intestine, and hypothalamus were sampled as above described. Tissues were kept at −80 °C and plasma at −20 °C until analysis.

### 4.4. Analytical Techniques 

#### 4.4.1. Biometric Parameters

Body weight gain and standard length gain were calculated as the percentage of bw or length relative to initial bw or length. Specific growth rate was determined by $\mathrm{SGR}=\left[\ln\left(W_{i}-W_{f}\right)/t\right]\times 100$, where $W_{i}$ and $W_{f}$ are initial and final body weights, and t = 7 days. 

#### 4.4.2. Locomotor Activity Recordings

Daily locomotor activity was recorded during the subchronic SR9009 treatment as previously described [41,55]. In short, infrared photocells (Omron Corporation E3S-AD12, Kyoto, Japan) were fixed onto aquaria walls, two of them below the automatic feeder and four placed at 3–9 cm above the bottom, one on each aquaria wall. Photocells emit infrared light and register pulses, every time the beam is crossed, into an actimeter and data-acquiring software (Adq16, Micronec, Madrid, Spain). Tanks were covered with opaque paper to avoid visual interferences. Food-anticipatory activity was determined as the number of pulses registered during the 4 h period prior to daily food delivery (10 a.m.).

#### 4.4.3. Plasma and Liver Metabolites

Plasma concentrations of glucose, triglyceride, and fatty acid were determined using enzymatic and colorimetric methods using commercial kits (Spinreact, Girona, Spain for glucose and triglycerides; Fuji, Neuss, Germany for fatty acids) adapted to microplates [41]. Liver samples were homogenized by sonication in 7.5 vols. of ice-cooled 1 N potassium bicarbonate and centrifuged (4 min, 13,500× *g*, 4 °C). The supernatant was assayed for concentration of fatty acid, triglyceride, and glucose using commercial kits as described above for plasma samples. Liver glycogen was assessed using the method of Keppler and Decker [56]. Glucose obtained after glycogen breakdown (after subtracting free glucose levels) and tissue glucose concentration was determined with a commercial kit (Biomerièux, Grenoble, France). 

#### 4.4.4. Liver Enzymatic Activity

Samples were homogenized ultrasonically in an ice-cooled buffer containing imidazole (50 mM), 2-mercaptoethanol (15 mM), potassium fluoride (100 mM), EDTA (5 mM), EGTA (5 mM), and protease inhibitor (Sigma Chemical, Madrid, Spain). Then, homogenates were centrifuged (10 min, 900× *g*, 4 °C), and the supernatant was recovered to be used in enzyme assays, using a microplate reader INFINITE 200 Pro (Tecan, Männedorf, Switzerland) and 96-well microplates. The enzymatic reaction rates were determined by the increase or decrease in absorbance of NAD(P)H at 340 nm. For CPT-1 activity, it was the increase in 5,5′-dithiobisn (2-nitrobenzoic acid)-CoA (DTNB-CoA) complex at 412 nm. The reactions were started by the addition of homogenates (15 µL) at a pre-established protein concentration, without the substrate in control wells (final volume 265–295 µL) and allowing the reactions to proceed at 20 °C for pre-established time periods (3–10 min). 

Enzyme activities are expressed in terms of mg protein, which was assayed using 96-well microplates following the bicinchoninic acid method with bovine serum albumin (Sigma Chemical, Madrid, Spain) as standard. The activities of GCK (EC 2.7.1.2), HK (EC 2.7.1.1), PFK (EC 2.7.1.11), PK (EC 2.7.1.40), G6Pase (EC 3.1.3.9), FBPase (EC 3.1.3.11), PEPCK (EC 4.1.1.32), G6PDH (EC 1.1.1.49), GSase (EC 2.4.1.11), GPase (EC 2.4.1.1), ACLY (EC 4.1.3.8), FAS (EC 2.3.1.85), and CPT-1 (EC 2.3.1.21) were assessed as previously described [41,57].

#### 4.4.5. Gene Expression Analysis

mRNA abundance of *npy*, *pomc*, *cartpt-I*, *hcrt*, *ghrelin*, *leptin-aI*, and *pepck* was determined by RT-qPCR as previously described [41,55], with minor modifications. Tissues were mechanically homogenized, and RNA was isolated from the liver, hypothalamus, and intestine using the trizol/chloroform method (TRI^®^ Reagent, Sigma Chemical, Madrid, Spain), and treated with RQ1 RNAse-Free DNAse (Promega, Madison, WI, USA). Then, 0.5 µg of total RNA was reverse-transcribed into cDNA in a 20 µL volume using random primers (Invitrogen, Waltham, MA, USA), RNAse inhibitor (Promega, Madison, WI, USA), and SuperScript IV Reverse Transcriptase (Invitrogen, Waltham, MA, USA). Samples were measured by duplicate by adding 1 µL of cDNA to iTaq TM Universal SYBR Green Supermix (Bio-Rad Laboratories, Hercules, CA, USA) in 96-well plates, with a final concentration of 0.5 µM of each forward and reverse primer, in a final volume of 10 µL. Each plate included a standard cDNA curve and water as a negative control. 

The RT-qPCR protocol included an initial denaturation step of 95 °C for 30 s, followed by 40 cycles of a two-step amplification program (95 °C for 5 s, and 60 °C for 30 s). Melting curves were monitored systematically to confirm the reaction specificity (gradient of 0.5 °C/5 s from 70 to 90 °C). GenBank reference numbers and primer sequences (Sigma-Aldrich, St. Louis, MO, USA) utilized for target and reference genes are shown in Table 1. The 2^−ΔΔCt^ method [58] was used to determine relative gene expression (fold change). Data obtained were relativized to the control group in each experiment. 

### 4.5. Statistical Analysis

Data are expressed as mean + SEM. For all statistical analysis, Sigmaplot^®^ software was employed. In order to confirm normality and homoscedasticity of data sets, Shapiro–Wilk and Levene tests were used, respectively, and if needed to meet these requirements, data were transformed to a logarithmic or square root scale. To compare data means from control and SR9009 groups, Student’s *t*-test was performed, considering the significance thresholds * *p* < 0.05, ** *p* < 0.01, *** *p* < 0.001.

## Figures and Tables

**Table 1:** Primers used in the RT-qPCR for housekeeping and target genes.

Gene	Access Number (GeneBank)	Sense	Sequence	Product (bp)
*actb*	AB039726.2	Forward	CGGGAGTGATGGTTGGCA	168
		Reverse	AACACGCAGCTGTTGTAGA	
*eef1a1*	AB056104	Forward	CCCTGGCCACAGAGATTTCA	101
		Reverse	CAGCCTCGAACTCACCAACA	
*npy*	M87297	Forward	TTCGTCTGCTTGGGAACTCT	151
		Reverse	TGGACCTTTTGCCATACCTC	
*pomc*	AJ431209	Forward	CTCACCACTGACGAGAACATCTTG	121
		Reverse	CGGTTTGCTCCAGCTCAGA	
*cartpt-I*	AY033816	Forward	GTGCCGAGATGGACTTTGAC	97
		Reverse	AGCTGCTTCTCGTTGGTCAG	
*hcrt*	DQ923590.1	Forward	ACTGCACAGCCAAGAGAGTTCA	166
		Reverse	GTTATTAAAGCGGCCGATATGC	
*ghrelin*	AF454389	Forward	TTCATGATGAGTGCTCCGTTC	124
		Reverse	GTCAGAATTCAAGTGGCGAATC	
*leptin-aI*	FJ534535.1	Forward	AGCTCCTCATAGGGGATC	192
		Reverse	TAGATGTCGTTCTTTCCTTA	
*pepck*	MK598557	Forward	TAACTGGCGTCATGGTGTGT	120
		Reverse	TAGCCGAAGAAAGGACGCAT	

## Data Availability

Data are contained within the article or supplementary material.

## Supplementary Materials

The following supporting information can be downloaded at: https://www.mdpi.com/1422-0067/23/6/2921/s1.
