# Supplementary material for: REV-ERBα Agonist SR9009 Promotes a Negative Energy Balance in Goldfish
Source: Int J Mol Sci. 2022 Mar 8;23(6):2921. doi: 10.3390/ijms23062921 (PMC8955992; doi:10.3390/ijms23062921)
Supplement: Supplementary file 1 [file ijms-23-02921-s001.zip › ijms-1608936-supplementary.pdf]

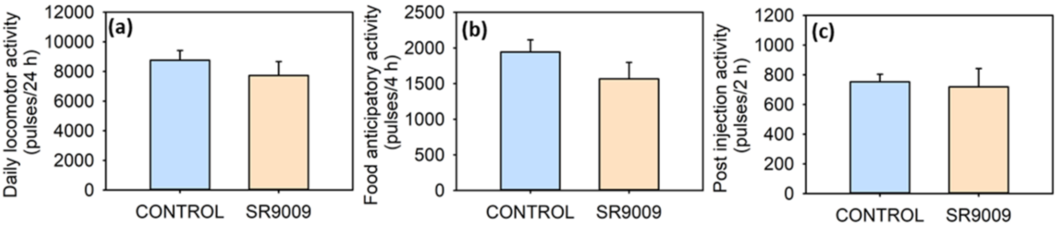

Figure S1. Effect of subchronic SR9009 treatment over locomotor activity of goldfish (*Carassius auratus*). Daily (**a**), food anticipatory (**b**), and 2 h post injection (**c**) activity recorded on the duration of the experiment (7 days) in both experimental groups. Data are expressed as mean + S.E.M (n = 12/group).

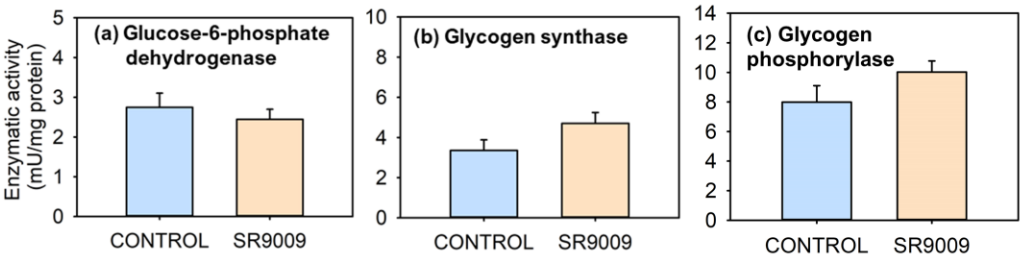

Figure S2. Effect of subchronic SR9009 treatment on liver activity of glucose-6-phosphate dehydrogenase (**a**), glycogen synthase (**b**), and glycogen phosphorilase (**c**) in goldfish (*Carassius auratus*) treated with vehicle alone (CONTROL) or containing SR9009 (100 μg/g bw). Data are expressed as mean + S.E.M. (n = 12/group).

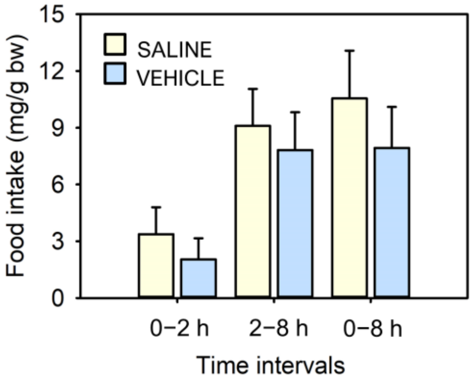

Figure S3. Effect of SR9009 vehicle on food intake in goldfish (*Carassius auratus*) in the intervals of 0-2, 2-8, and 0-8 h after acute IP injection of saline solution alone or containing Kolliphor (15%). Data are expressed as mean + S.E.M. (n = 9 fish/group) .
